# Interventions to Improve Depressive Symptoms in Community-Dwelling Older Adults: A Scoping Review

**DOI:** 10.1007/s10935-026-00911-z

**Published:** 2026-05-28

**Authors:** Laura Restrepo-Escudero, María Alejandra Jaimes Velásquez, Isabela Arango, Sofía Santos-Simancas, Valentina Ramírez Castellanos, Daniel Uribe, Jenny Patricia Muñóz, Lina María González-Ballesteros

**Affiliations:** 1 Department of Psychiatry and Mental Health, Faculty of Medicine, Pontifical Xavierian University, Cra. 7 #40 - 62, Bogotá, Colombia; 2 Mental Health and Resilience Research Seedbed, Department of Psychiatry and Mental Health, Faculty of Medicine, Pontifical Xavierian University, Cra. 7 #40 - 62, Bogotá, Colombia

**Keywords:** Mental health, Older people, Public health strategies, Public services

## Abstract

Depression is a prevalent psychiatric disorder among adults aged 65 and older, significantly impacting their well-being. With an aging global population, effective interventions delivered in community settings are vital to combat this issue. This review provides an overview of the characteristics of interventions addressing depression in community-dwelling older adults, identifying knowledge gaps by synthesizing current data. The search strategy entails a systematic database search across PubMed, EMBASE, Ovid, and PsycNet, without any time or language restrictions. Studies were included if they reported interventions delivered in community settings that aimed to improve depressive symptoms or reduce the prevalence of depression in older adults. A total of 20 studies met the inclusion criteria. Interventions were grouped into four main types (discussion groups, individual contact, social engagement activities, and skills courses), often combined. Most reported short-term reductions in depressive symptoms, but evidence was limited by small samples, reliance on self-reported outcomes, and scarce long-term follow-up. Interventions delivered in community settings show promise for supporting older adults’ mental health, especially when they are adapted to local needs. Improving recruitment strategies and tackling cultural and logistical barriers could expand their reach and accessibility, and strengthen the contribution of these programs alongside specialist mental health care.

**Supplementary Information:**

The online version contains supplementary material available at https://doi.org/10.1007/s10935-026-00911-z.

## Introduction

A significant social challenge of the twenty-first century is the aging of the world’s population. This will affect employment and financial markets, demand for housing, transportation, social security, family dynamics, and relationships between generations (Nguyen & Vu, 2013). The proportion of adults 65 years and older is rapidly increasing, with projections indicating a rise from 10% in 2022 to 16% in 2050 (United Nations, n.d.). It is also predicted to surpass the population of children under age 5 by 2050 and be nearly equal to children under age 12 (United Nations, n.d.). These trends make it increasingly important to address health issues common in later life, including mental health conditions such as depression.

Depression is a prevalent mental health disorder among older adults, but its epidemiology in late life is complex. Prevalence estimates in community and clinical samples vary from 1% to 16% for major depression, 2% to 19% for minor depression, and 7.2% to 49% for clinically relevant depressive symptoms (Cai et al., 2023; Hu et al., 2022; (Jalali 2024). A recent meta-regression suggests an overall increasing trend in depression prevalence with age among older adults (Han et al., 2021). Various factors are associated with a higher prevalence of depression, such as poor physical function, being without a partner and lower levels of education (Sjöberg et al., 2017); (Sivertsen 2015).

Recent epidemiological studies report that, while prevalence may remain stable or slightly increase in the ‘younger-old,’ rates are significantly higher among individuals with chronic illnesses, functional limitations or those living in institutional settings (Giebel et al., 2022; Hu et al., 2022). At the same time, annual cumulative incidence of depressive symptoms in community-dwelling older adults ranges from 1.3 to 26.6% (Brasileiro et al., 2024). This highlights the importance of preventive actions and early care interventions in community settings.

In response to the growing burden of late-life depression, a range of interventions have been developed for delivery at the community level. Community-based interventions are typically described as multi-component and contextually adapted efforts, engaging community members during design and implementation (Brunton et al., 2017). Key components include involvement of multiple stakeholders (e.g. community leaders, organizations, healthcare providers), empowerment-focused approaches and assessing local needs (Castillo et al., 2019; Mikkelsen et al., 2016). These programs offer advantages such as wider outreach and reduced reliance on specialist healthcare systems. However, in the late-life depression literature many programs delivered outside inpatient or residential settings incorporate only some of these features. In this review we therefore use the broader term ‘interventions delivered in community settings’ and later examine to what extent they incorporate elements of community-based approaches.

A variety of strategies have been developed in community settings. Programs that encourage physical activity in seniors, such as group fitness classes or individualized exercise programs, are associated with improved mood and reduced depression symptoms (Giebel et al., 2022). Social isolation is a significant risk factor for depression in older adults, which can be mitigated through interventions promoting social interactions through group activities, volunteer work, or social support groups (Gardiner et al., 2018). Interventions combining multiple components, such as social engagement, exercise, and cognitive stimulation, tend to be more effective in reducing depression than single-component interventions (Porteny et al., 2020).

Psychological interventions provide another important tool for community settings. Cognitive behavioral therapy (CBT) groups adapted for delivery outside specialized services, not only address individual cognitive distortions and maladaptive behaviors but also promote social interaction and peer support, which could reduce the sense of isolation common among older adults (Corpas et al., 2022; Motamed-Jahromi & Kaveh, 2021; Reynolds et al., 2022). Guided self-help or bibliotherapy programs based on CBT principles can enhance self-management abilities and improve mental health outcomes (Corpas et al., 2022). These findings suggest that diverse types of programs have been developed for delivery in the community, but their content and implementation strategies vary widely.

In light of these considerations, this scoping review aims to provide an overview of interventions delivered in community settings to improve depressive symptoms in community-dwelling older adults, describing their features, activities, delivery and dosage, evaluating to what extent they incorporate features commonly associated with community-based approaches. We synthesized the reported effectiveness of these programs and identified the main implementation determinants (facilitators and barriers). The review does not aim to identify a single best way to deliver these interventions, but rather to offer an overview of the range of approaches described in the literature.

## Methodology

### Overview

This paper was conducted following the JBI methodology for scoping reviews (Peters et al., 2020) and the PRISMA-ScR (Preferred Reporting Items for Systematic Reviews and Meta-Analyses extension for Scoping Reviews) guidelines, provided as Supplementary material (Tricco et al., 2018). A scoping review was conducted of interventions delivered in community settings (e.g. primary care, community mental health services, senior centers, community organizations or participants’ homes) that have been developed for combating depression in adults 65 years and older. These include discussion groups, peer outreach programs, skill courses, and psychotherapeutic interventions, among others. In addition to describing the characteristics of these interventions (features, activities, mode, and frequency of delivery), mental health outcomes of interest include changes in depressive symptoms or in depression prevalence in the older population in community settings. A review protocol was prepared for the guidance of the authors and was registered in the Open Science Framework: https://doi.org/10.17605/OSF.IO/MGCPZ.

### Eligibility Criterias

All types of quantitative and qualitative studies were included. Interventions were not required to meet all criteria of classical community-based programs (e.g. co-design with community members, multi-level components). Instead, we focused on interventions delivered outside healthcare settings (e.g. nursing homes or hospitals) that aimed to improve depressive symptoms and/or decrease the prevalence of depression in older adults, regardless of whether the outcome was negative. Information sources were restricted to community settings from any geographic region and cultural background. Since this study aims to determine the effectiveness of interventions on depression as a primary psychiatric disorder, studies performed on patients with cognitive impairment, psychosis, and/or psychotic disorders were excluded. Opinion papers and unpublished studies were excluded. Finally, studies that lacked a quality review process and were not available in full text or a suitable format for analysis were excluded.

### Information Sources

The information sources included peer-reviewed journals and preprint articles. We conducted an initial search in the PubMed database to find relevant articles on community interventions targeting depression in older adults. We modified the keywords and index terms from the first search’s titles and abstracts to develop a customized search strategy for each database included. Second, we conducted a systematic search of peer-reviewed and preprint literature in PubMed, EMBASE, Ovid, and PsycNet, without any language or time restrictions, from inception to July 2024, identifying sources reporting on mental health outcomes of interest, such as depressive symptoms or prevalence of depression. We reviewed articles that emerged from these searches and relevant references they cited. We included articles published in English and Spanish.

### Search Strategy

The search of studies reporting interventions for combating depression in community-dwelling older adults was performed in PubMed, EMBASE, Ovid, and PsycNet using the following search strategy (shown as constructed in PubMed) without any time or language restrictions:

(((“Aged“[Mesh]) OR (elderly OR “older adults” OR “older people”)) AND ((“Depression“[Mesh] OR “Depressive Disorder“[Mesh] OR “Depressive Disorder, Major“[Mesh]) OR (depres* OR “depressive disorder”)) AND ((“Community Mental Health Services“[Mesh]) OR (“community-based intervention” OR “community-based interventions” OR “community intervention” OR “community interventions”)) (See Appendix 1 for the complete search strategies).

### Selection of Sources of Evidence

All the included articles were uploaded into Rayyan QCRI software for duplicate removal. The titles and abstracts were screened by two reviewers (LRE, MAJ) to identify relevant studies. The full text of selected sources was assessed by seven reviewers (LRE, MAJ, IA, SS, OG, DU, VR) for eligibility as part of the scoping review according to inclusion criteria. Disagreements between reviewers were resolved with an additional reviewer (LMG).

### Data Charting Process

Data collected from studies retrieved through the systematic search were extracted by the reviewers using piloted forms into a Microsoft Excel spreadsheet for analysis. Data charting was done independently and reviewed by one of the investigators (LRE).

### Data Items

Data items extracted from all information sources regarding study characteristics included study design, context (e.g., target population, country, ethnicity, and setting), and sample characteristics (e.g., size, age, gender, and marital status). As to the interventions, data items sought program characteristics in terms of components, activities, mode, and doses of delivery (referred to as frequency and duration). We classified and grouped the interventions based on the services they provided. Mental health outcomes data items included tools for screening and diagnosing depression and/or depressive symptoms, along with any changes in the outcome following the implementation of the intervention. These changes were measured through differences in total questionnaire scores or by evaluating clinical progress through structured clinical interviews.

We extracted any information that authors reported on factors that facilitated or hindered the implementation of the interventions (e.g. recruitment, adherence, delivery). Data was mainly drawn from the results and discussion sections of the included papers. We performed an inductive thematic content analysis: two reviewers independently coded reported determinants, grouped similar codes into categories (e.g. ‘financial support’, ‘local staff’, ‘ability to tailor the intervention’), and then classified each category as facilitator, barrier, or both, based on how it was described across studies. Disagreements were discussed until consensus.

### Critical Appraisal of Individual Sources of Evidence

The methodological quality of included studies was evaluated using the Critical Appraisal Skills Programme (CASP) checklists (CASP, 2023). For each study, we selected the checklist that best matched its design, which consists of structured questions covering internal validity, clarity of reporting and the applicability of the findings. Two reviewers independently completed the relevant CASP checklist for each study and summarized the main strengths and limitations in a brief narrative appraisal, identifying common methodological issues across studies which are referenced throughout the results section.

### Synthesis of Results

We grouped the studies based on the type of intervention under evaluation. Information regarding the interventions’ characteristics was tabulated and summarized in the text. Categorized determinants associated with the implementation of different programs were identified for individual studies and outlined in tables and text. Quantitative meta-analysis was not performed due to high heterogeneity among studies.

## Results

### Selection of Sources of Evidence

**Fig. 1:**
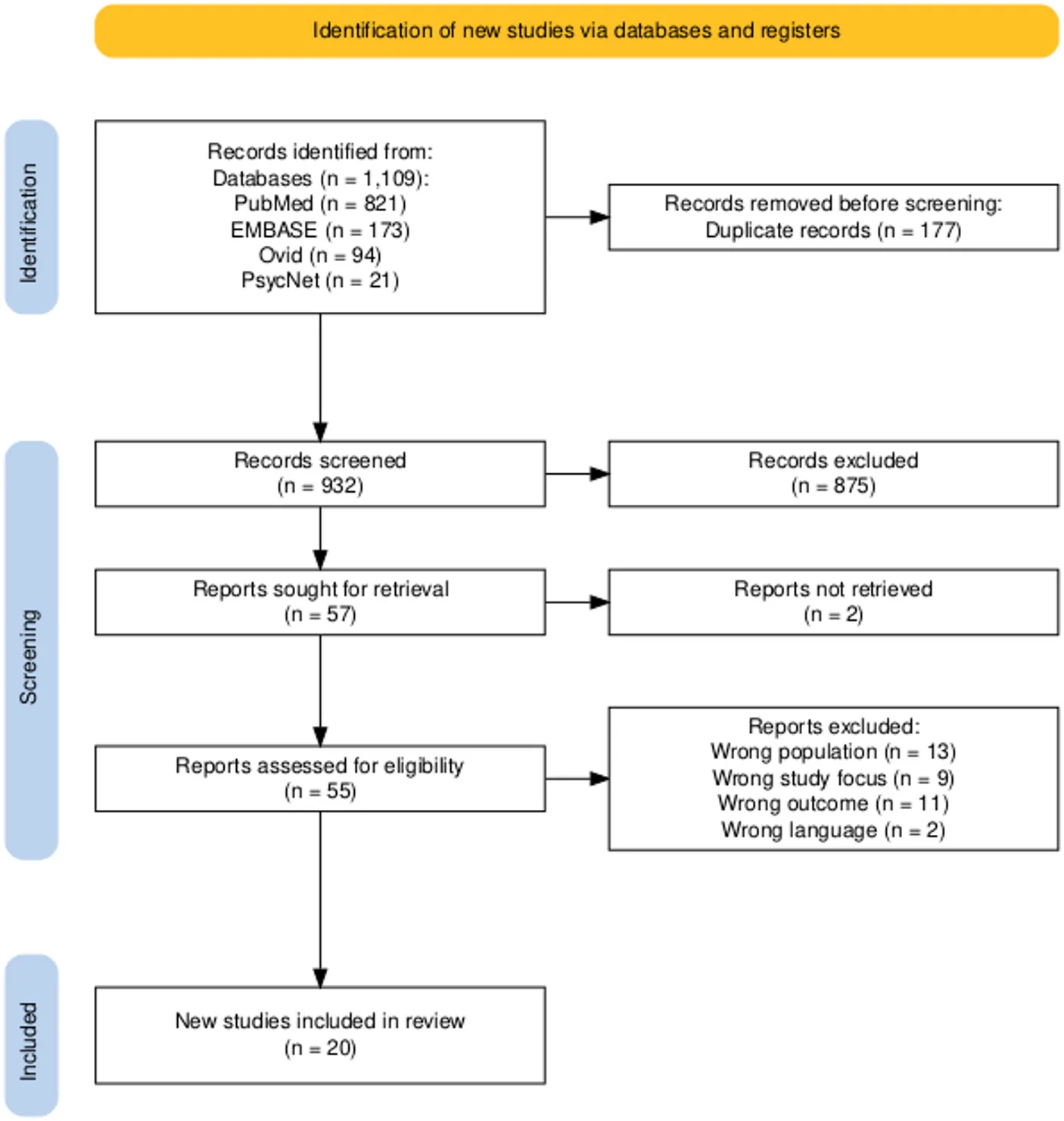
A flow diagram for selecting evidence sources

The systematic search for interventions addressing depression in community settings identified 1109 records, of which 932 were screened after removing duplicates. We included 57 studies for full-text screening after excluding 875 papers based on title and/or abstract. However, two articles (abstracts from scientific posters and unavailable journals) did not have full text, which left 55 final studies awaiting eligibility assessments. 13 studies were not conducted exclusively on adults aged 65 years and older or included participants without dementia or psychosis; among the remaining papers, nine had an inadequate study focus (wrong setting or intervention), 11 articles did not report measures of depression, and two were in languages unknown to authors (Chinese and Japanese), resulting in the inclusion of 20 studies (Fig. 1) (Haddaway et al., 2022).

### Characteristics of Individual Sources of Evidence

Reviewed studies include a variety of approaches aimed at older adults, using methods in different cultural and geographical contexts. The characteristics of the 20 studies reviewed are presented in Table 1. Most studies were conducted in the United States. They employed various methodological designs, including mixed-methods longitudinal studies, randomized pilot trials, systematic reviews, and quasi-experimental cohort studies, with half of the studies (*n* = 10) designed as randomized controlled trials (RCT). Researchers conducted studies in diverse cultural, social, and geographical contexts, including urban settings with low-income older adults (Kotwal et al., 2021) and rural areas in South Korea (Oh et al., 2020). The samples vary in size, age, gender, and marital status. Populations included older adults from different ethnicities (African American, Latin, Asian, Black, Indigenous, Caucasian, Hispanic), and the number of participants ranged from 6 to 1200. The studies were published between 1991 and 2023.

**Table 1:** Characteristics of the studies reviewed

Study ID	Aim of the study	Study design	Data collection period	Population Description	Country	Method of recruitment	Participants (*n*, age, gender, marital status)	Summary of CASP appraisal
Sirey, 2021	To test the feasibility, acceptability, and impact of PROTECT, a short-term, community-based psychotherapy designed for older adults who are victims of elder abuse and with depression.	Randomized controlled trial	9 weeks	Elderly adults who were victims of elder abuse and displayed depressive symptoms	United States	Elder abuse service providers.	*n* = 40; mean age = 71; 81·3% female; marital status: 38% widowed, 22·6% married, 16·7% single, 14·7% divorced, 8% separated	Clear RCT with validated outcomes; no long-term follow-up limits understanding of sustained effects, with a small sample size (no calculation reported), unknown psychosocial impact and cost-effectiveness
Cuijpers, 1998	To evaluate the effectiveness and dropout rates of psychological outreach programs aimed at treating depression among elderly individuals in community settings.	Meta-analysis	Not reported.	Studies regarding the effects of a psychological intervention specifically aimed at older adults.	Not reported	Database search	*n* = 14 studies; age between 55 and 83; predominantly female (55–98%); marital status: not reported	Clear focus and outcomes, most of the studies selected had small sample sizes or no control group, not enough information to tell if results could be applied locally.
Blanchard, 1995	To assess the impact of community nurse management on older adults identified as depressed in comparison to standard general practitioner (GP) care.	Randomized controlled trial	6 months	Older adults with probable pervasive depression who lived at Gospel Oak in London	United Kingdom	Household enumeration	*n* = 112; mean age = 76·3; 83% female; marital status: not reported	Clear RCT with validated outcomes, with a comparable control group, but no sample size calculation reported, no long-term follow-up and with limitations surrounding the screening tools used
Kotwal, 2021	To evaluate the effectiveness of a peer outreach intervention in reducing loneliness, improving social well-being, and addressing behavioral health needs in low-income, community-dwelling older adults of diverse racial and ethnic backgrounds.	Mixed-method, two-year longitudinal study	2 years	Low-income older adults, primarily living independently in community settings	United States	Community-based organizations.	*n* = 74; mean age = 74; 58% male, 42% female; marital status: not reported	Clear mixed-methods design and relevant outcomes, but small sample with no sample size calculation reported and short follow-up limit generalizability
Joo, 2025	To evaluate the effectiveness of an 8-week community-based intervention using peer mentors to provide social support and self-care skills to older adults with depression.	Randomized controlled trial	8 weeks	Low-income older adults with depression	United States	Local community health centers	*n* = 149; mean age = 70; 84.3% female; 84.3% not married	Clear RCT with validated outcomes and a well-defined population; however, implementation challenges, target sample not achieved (*N* = 160), loss to follow-up, and lack of blinding limit the generalizability of findings
Sadler, 2018	To investigate the effectiveness of cognitive behavioral therapy in treating insomnia and depression among older adults within community mental health services.	Randomized controlled trial	8 weeks	Older adults who are experiencing both insomnia and depression, all receiving care in community mental health services	Australia	Local community health services	*n* = 72; mean age = 75; 56% female, 44% male; marital status: 40% married, 28% widowed, 15% divorced, 6% separated, 7% single, 4% defacto	Clear RCT with validated measures and comparable groups; concerns about lack of blinding, unadjusted confounders, sample size calculation not reported and short follow-up
Mastel-Smith, 2007	To evaluate the effectiveness of a psychosocial intervention, the Life Story Workshop, on improving depressive symptoms in older adults	Randomized controlled trial	10 weeks	Older adults experiencing depressive symptoms	United States	Local community services	*n* = 31; mean age = 70·12; 81% female; marital status: predominantly married (not reported)	Clear purpose and consistent delivery, but very small sample (no calculation reported) and lack of randomization limit strength of the findings
Husaini, 1990	To evaluate the effectiveness of a therapeutic health program for Black elderly individuals living in subsidized high-rise apartments in Nashville, Tennessee	Randomized controlled trial	6 weeks	Older black adults living in low-income housing	United States	Local community housing projects	*n* = 125; mean age = 72·3; 75% female; marital status: 4·8% married, 95·2% not married	Clear RCT with validated measures and comparable groups; concerns about sample size calculation not reported, no clear long-term outcomes, only hypothesis, and an intervention group with mildly specific characteristics
Galinha, 2023	To evaluate the short and long-term effects of a community-based group singing intervention on older adults’ perceived physical and mental health.	Randomized controlled trial	6 months	Older adults from community settings	Portugal	Local community organizations	*n* = 149; mean age = 76·7; 82·4% female; marital status: 45·3% widowed, 28·7% married, 13·7% divorced, 12·3% single	Clear RCT with validated outcomes, strong retention, target sample achieved (*N* = 140) and adjusted analysis conducted, but no long-term follow-up limits understanding of sustained effects
Paquet, 2023	To conduct a meta-review to evaluate social prescription interventions designed to address social isolation and loneliness among older adults. It also integrated real-world, on-the-ground resources from community services to enhance the applicability of these interventions in practice.	Meta-review	Not reported	Adults aged 60 years and above, who were experiencing or at risk of social isolation and loneliness	Not reported	Database search	*n* = 67 studies; Age: ≥ 50; Gender and marital status not reported	Broad and relevant review, but unclear quality assessment and high heterogeneity limit confidence in the findings
Sirey, 2012	To evaluate the feasibility and preliminary effectiveness of the ACTIVATE intervention	Pilot study	12 weeks	Older adults currently in treatment for depression but still exhibiting symptoms of the condition	United States	Home meal service programs	*n* = 43; mean age 77·5; 76·7% female; marital status: not reported	Clear pilot with validated outcomes; limited by no control group, no blinding, no sample size calculation reported and short follow-up
Oh, 2020	To evaluate the feasibility and effectiveness of a 12-week village-based intervention aimed at reducing depressive symptoms and suicidal risk among older adults in rural areas.	Randomized feasibility study	12 weeks	The study involved older adults from two rural villages in Yeoncheon County, South Korea	South Korea	Local community health services	*n* = 160; mean age = 74·2; 55·8% female; marital status: not reported	Feasibility RCT with validated outcomes, strong retention and target sample achieved (*N* = 160); limited by no blinding, no analysis of confounding variables and lack of long-term follow-up
Van Citters, 2004	To systematically review the effectiveness of community-based mental health outreach services for older adults, focusing on their impact on mental health outcomes, accessibility, and service utilization.	Systematic review of randomized controlled trials and cohort studies.	Varied from 3 months to 3 to 4 years	Older adults receiving community-based mental health services	Not reported	Database search.	*n* = 12 studies; age between 75 and 85; predominantly female; marital status: predominantly widowed or lived alone	Results were precise and answered a clear research question; search strategy could include more studies and quality assessment should be done
Van ‘t Veer-Tazelaar, 2011	To assess the feasibility and effectiveness of a stepped-care program in preventing anxiety and depression among elderly community residents at high risk for these conditions.	Randomized controlled trial	2 years	Elderly individuals living in the community and at high risk of developing anxiety and depression	Netherlands	Primary care practitioners	*n* = 170; mean age = 81·4; 74% female; and 30% married	Study design fulfilled all criteria for RCT and results were completely detailed; blinding was not possible; target sample size achieved (65 participants per group); unknown cost, adverse events, and generalizability
Knight, 2017	To evaluate the effectiveness of a geriatric mental health day treatment service in improving mental health outcomes among older adults with mood disorders.	Interventional study (before- after study)	10 weeks	Older adults experiencing mood disorders, such as depression and anxiety, who were living in the community	Canada	Primary care providers, geriatricians, or other health professionals.	*n* = 255; mean age = 73·2; 67·6% female; marital status: 50·9% married, 23·3% divorced/separate, 22·6% widowed, 3·1% never married.	Clear focus and outcomes, possible selection bias, no sample size calculation reported, no analysis of confounding factors, unknown long-term effects and generalizability
Guirguis-Younger, 2008	To pilot and refine a community-based behavioral intervention designed to treat depression in seniors	Longitudinal across-subjects study	6 weeks	Underserved older persons with medical conditions and depressive symptoms	Canada	Local community health services	*n* = 6; mean age: 74; 4 males; marital status: 1 unmarried, 1 widow, 3 married, 1 not reported	Clear focus and outcomes, possible selection bias, basic analytic models with small sample size (no calculation reported), unknown long-term effects and generalizability
Rodríguez-Romero, 2021	To assess the effectiveness of a community-based intervention in reducing perceived loneliness, depression, and increasing social support and quality of life in older adults.	Randomized controlled trial without blind evaluation	6 months	Older adults from an urban area of Barcelona, who were identified as experiencing loneliness	Spain	Family physicians and nurse practitioners	*n* = 55; mean age = 80·2; 87·3% female; marital status: 21·8% married, 70·9% single, 7·3% widowed	Clear RCT with validated measures and complete follow-up; concerns about non-blinded sample and target sample not achieved (40 participants per group); unknown long-term effects, cost, adverse events, and generalizability
Carandang, 2020	To assess the effectiveness of 3-month-duration interventions with peer counseling, social engagement, and combination vs. control in improving depressive symptoms of community-dwelling Filipino senior citizens.	Open (non-blinded), non-randomized trial	3 months	Senior citizens from Muntinlupa City, identified as at risk for depression	Philippines	Home visits by barangay health workers.	*n* = 264; mean age = 68·3; 70·8% female; marital status: 46·7% married, 40·7% widowed, 9·4% never married, 3·2% separated	Clear study with validated measures and comparable groups, target sample size achieved (60 participants per group), concerns about non-randomized, unblinded, and no intention-to-treat, unknown long-term effects, adverse events, and cost
Reid, 2019	To evaluate the feasibility, safety, and effectiveness of translating the structured physical activity interventions from the original Lifestyle Interventions and Independence for Elders clinical trial into a real-world community-based setting for older adults at risk of mobility-disability.	Randomized controlled trial	24 weeks	Older adults with severe lower extremity functional limitations	United States	Community-based senior centers	*n* = 40; mean age = 77; 85·3% female; marital status: not reported	Clear study with clear question, balanced groups, meaningful improvements, unblinded study, small sample size (no calculation reported), incomplete intention-to-treat, unknown psychosocial impact and cost-effectiveness
Bartsch, 2009	To compare the outcomes of older adults served by the Senior Reach program with those served by the Spokane Gatekeeper program in terms of mental health, functioning, and social isolation	Comparative observational study	Twenty-eight months	Older adults at risk for social isolation and emotional disturbance	United States	Community members	*n* = 226; mean age = 76·6; 71·7% female; marital status: 26·7% married, 26·6% divorced/separate, 42% widowed, 4·7% never married	Clear study with validated outcome measures, concerns about confounders, sample size calculation not reported, unequal follow-up, unknown adherence details

### Characteristics and Effectiveness of Interventions

The 20 studies included were grouped according to the intervention delivered, and their characteristics were described in Table 2.

**Table 2:** Characteristics of the different types of interventions identified

Type of intervention	Main components and activities	Delivery mode	Dose described (frequency and duration)	Depression measure(s)	Main outcome on depressive symptoms
Personal contact (4 studies/1 review) Sirey, 2021 Cuijpers, 1998 Blanchard, 1995 Kotwal, 2021 Joo, 2025	One-to-one supportive contacts such as home visits, telephone calls, brief psychological interventions	Individual, face-to-face visits and/or phone calls delivered by clinicians, peers or trained volunteers	Home visits or regular scheduled contacts over several months (e.g., ~ 8–10 weekly visits of ~ 45 min over three months) according to participants’ need and interest	PHQ-2 and PHQ-9; Montgomery-Åsberg Depression Rating Scale (MADRS); GMS diagnostic criteria; a range of depression scales across studies included in the meta-review	Meta-analytic evidence (Cuijpers, 1998) showed interventions had large effects on depressive symptoms (dctr 0.77). Individual trials reported greater reduction in depression scale scores and higher remission rates compared to control groups, although Joo et al. did not find between group differences. Kowtal et al. found the proportion of participants screening positive for depression decreased
Discussion groups (3 studies/reports) Sadler, 2018 Mastel-Smith, 2007 Husaini, 1990	Group sessions focused on discussing personal experiences (e.g. life stories), coping strategies and psychoeducation about feelings and aging	Face-to-face group sessions (≈ 10 participants), led by trained instructors	Weekly sessions over several weeks (e.g. 6–11 weeks) of 60–90 min per session, as reported in the original trials	Geriatric Depression Scale (GDS); BSI-18 (psychological distress); NIMH Center for Epidemiologic Studies Depression scale (CES-D)	All three trials reported reductions in depressive symptoms over time. In Sadler et al., depression severity improved in both CBT-based sessions (*p* < 0.001), with follow-up remission rates between 64–73%; in other studies, participants in discussion groups had significantly fewer depressive symptoms at follow-up compared with baseline
Social engagement (1 study/1 review) Galinha, 2023 Paquet, 2023	Group-based social activities, recreational activities (e.g. singing sessions) and engagement in community programs designed to increase social participation	Face-to-face group activities in community settings	Multi-week group programs (e.g., ~ 34 two-hour sessions over 4 months)	The Positive Affect and Negative Affect Schedule in the group-singing trial; a range of depression scales across studies included in the meta-review	Galinha, et al. identified significant indirect effects on perceived depression levels, through higher positive affect scores measured with The Positive Affect and Negative Affect Schedule. The meta-review reported overall reduction in depressive symptoms, although effect sizes and consistency varied across studies
Skill course (1 study) Sirey, 2012	Courses aimed at developing participants’ personal skills (e.g. problem-solving, psychoeducation about depression, motivational techniques)	Face-to-face sessions led by trained social workers or similar professionals.	At least two 30-min sessions with lectures, role-play and similar strategies	Structured Clinical Interview for DSM-IV Axis I Disorders for diagnosis; MADRS for severity	The primary outcome was improvement in depression management (e.g. treatment changes and adequacy); most participants took steps to change or optimize their treatment (66.6%). A general reduction in depression severity was observed when comparing MADRS scores from baseline and the week 12 follow-up
Discussion groups and personal contact (1 study/1 review) Oh, 2020 Van Citters, 2004	Programs combining group discussions with individual contacts (e.g., psychoeducational groups and workshops plus home visits or telephone calls)	Mix of group sessions (≈ 10 to 12 participants), and one-to-one contacts; delivered by multidisciplinary staff (e.g., social workers, nurses, care managers, physicians and residential staff)	Programs ranging from 12 weeks to 4 years, frequency of individual contacts varied according to individual risk (usually weekly)	Korean version of the GDS; Korean version of the Composite International Diagnostic Interview; a range of depression scales across studies included in the meta-review	A greater decrease was observed in the intervention group than in the control, although the difference did not reach significance (*p* = 0.296). The systematic review reported overall reduction in depressive symptoms, although effect sizes and consistency varied across studies
Skill course and personal contact (4 studies) Van ‘t Veer-Tazelaar, 2011 Knight, 2017 Guirguis-Younger, 2008 Rodríguez-Romero, 2021	Skill-building courses or workshops (e.g., stepped care, CBT-based components, bibliotherapy, mindfulness, psychoeducation) combined with individual contacts	Individual sessions (often home-based through self-administered tools) plus group or follow-up contacts, delivered by nurses, psychologists or other trained staff	Programs with one to three sessions per week, durations from 6 weeks to 2 years	CES-D; GDS; Hamilton Rating Scale for Depression; Yesavage	Interventions generally showed reduction in depressive symptoms at follow-up and fewer participants screening positive after interventions compared to controls. Secondary outcomes evaluated by Rodríguez-Romero et al. included improvement in the degree of perceived loneliness (*p* = 0.012) and increased social support (*p* = 0.002)
Multicomponent Carandang, 2020 Reid, 2019 Bartsch, 2009	Programs mixing several components (e.g., psychoeducation, social activities and events, case management, interactive games, gatekeeper training)	Combination of face-to-face group activities, individual contacts and/or telephone support; often involving community volunteers and health providers	Sessions once or twice a week, or several times per month; program lengths varied from 3 months up to 2 years	GDS-15 or GDS; CES-D	Depressive symptoms reduced after the intervention (*p* < 0.05), although in Bartsch et al. differences between groups were non-significant. In Carandang et al., the multicomponent combination group showed the largest effect on improving psychological resilience and reductions in depressive symptoms compared with controls (d = − 1.33)

#### Personal Contact/Peer Outreach

Personal contact interventions focus on social support through one-to-one contacts, which can be delivered in person or virtually. Weekly visits were delivered by social workers (Sirey et al., 2021), nurses (Blanchard et al., 1995) or trained community members (Kotwal et al., 2021; Joo et al., 2025), and usually lasted 30 to 60 min. Therapeutic oriented interventions included problem-solving therapy, supportive therapy and cognitive behavioral therapy (Cuijpers, 1998), but peer delivered approaches involved goal setting, self-care skills and companionship (Kotwal et al., 2021; Joo et al., 2025). Depression outcomes varied; Sirey et al., 2021 found reduction in depression severity, higher remission rates and improved quality of life, while Blanchard et al. (1995) and Kotwal et al. (2021) identified fewer cases of participants who screened positive for depression after interventions. Joo et al. (2025) did not identify depression score differences with the control group, but qualitative findings suggested the intervention group learned coping skills and experienced changes in mood; whereas the control group described primarily enjoyment from sharing a conversation amid social isolation of COVID-19.

#### Discussion Groups

Discussion groups focused around discussing personal experiences, coping strategies and psychoeducation about feelings and aging. Over several weeks, weekly sessions of around 60–90 min each were led by trained professionals, in a face-to-face setting. In the three trials that implemented discussion groups, there was a reported reduction in depressive symptoms over time, and participants in discussion groups had significantly fewer depressive symptoms at follow-up compared with baseline (Husaini et al., 1990; Mastel-Smith et al., 2007). When CBT-based sessions were implemented, depression severity improved with follow-up remission rates between 64 and 73% (Sadler et al., 2018).

One study and one review focused on interventions based on discussion groups and personal contact, in which group discussions strategies (psychoeducational groups or workshops) were implemented alongside individual contact (home visits or telephone calls). The frequency of individual contact varied according to individual risk, although they were delivered usually on a weekly basis (Oh et al., 2020). As a result, a greater decrease in depression related outcomes was observed in the intervention group, although this difference was not statistically significant (Oh et al., 2020). In the systematic review, the duration of the intervention varied from months up to 3–4 years, and all the interventions included were associated with significant improvement in depressive symptoms, although it is worth mentioning that effect sizes and consistency varied across studies included (Van Citters & Bartels, 2004).

#### Social Engagement

Social engagement interventions involved group-based social activities, support groups with educational elements, recreational activities and engagement in community programs designed to increase social participation. In the meta-review, there was an overall reduction in depressive symptoms with interventions targeting social interactions, promoting mental and physical well-being and improving social health through home and community care, although consistency varied across studies (Paquet et al., 2023). In a study where the primary intervention focused around group singing sessions over a four-month period, in a face-to-face setting, the primary outcomes described significant indirect effects on perceived depression levels, with higher positive affect scores on the Positive Affect and Negative Affect Schedule; however, it showed no direct effects on reduction of stress, anxiety or depression evaluated with the 21-item Depression, Anxiety, and Stress Scales (Galinha et al., 2023).

#### Skill Course

Only one study focused solely on skill course-based interventions, where courses aimed to develop participants’ personal skills were delivered in a face-to-face setting led by trained social workers or similar professionals, over at least two 30-min sessions. The intervention consisted of a brief, personalized assessment designed to identify barriers in depression treatment in order to help participants improve their treatment. The primary outcome showed that most participants took steps to change or optimize their treatment surrounding depression, and overall, a general reduction in depression severity was observed during follow-up when comparing Montgomery Asberg Depression Rating Scale scores from baseline (Sirey et al., 2012).

Four studies focused around skill course and personal contact, where participants could receive interventions based around psychoeducation and brief CBT sessions if needed, for an up to two year period (Van ‘t Veer-Tazelaar et al., 2011), or educational workshops which included mindfulness, yoga and walks in a face-to-face setting led by a lead nurse, over a period of six months (Rodríguez-Romero et al., 2021). In another study, the intervention lasted up to 10 weeks, and included various community outings and therapy sessions (Knight & Alarie, 2017). Overall, when skill courses and personal contact were implemented simultaneously, a reduction in depressive symptoms at follow-up could be observed; also, fewer participants screened positive after interventions compared to control groups. Secondary outcomes evaluated by Rodríguez-Romero et al. (2021) included improvement in the degree of perceived loneliness (*p* = 0.012) and increased social support (*p* = 0.002).

#### Multicomponent Programs

One study, an open (non-blinded), non-randomized trial had a multicomponent program involving peer counseling, social engagement and the combination of both, resulting in a significant improvement on geriatric depression scores after three months, finding the largest effect on the group receiving the combined strategies (Carandang et al., 2020). Another RCT involving exercise and social engagement activities, through biweekly 50-min workshops found greater improvements in quality of life and depressive symptoms after physical activity, although *p* values were not reported (Reid et al., 2019). A gatekeeper model combined with wellness programs, telephone contact and psychoeducation over a 28-months period found overall depression scores improve from baseline to discharge, but scores did not differ significantly between groups (Bartsch & Rodgers, 2009).

### Implementation Determinants: Facilitators and Barriers

Across the included studies, authors reported multiple factors that influenced how interventions were implemented. These determinants were grouped into 14 categories and synthesized in Table 3, with additional examples by study provided in Supplementary Table S1. They could either work as facilitators, barriers or both, depending on each setting. Overall, the most frequently described determinants were the ability to tailor the intervention, access to participants, involvement of local staff, participant engagement, staff training and supervision and the involvement of health care providers.

**Table 3:** Implementation determinants identified across studies

Determinant category	How it facilitated implementation	How it hindered implementation	Role (facilitator/barrier/mixed)	Number of studies (facilitator)	Number of studies (barrier)
Financial support	External or institutional funding covered expenses (e.g. staff time, training) allowing programs to be delivered and sustained over time.		Facilitator	3	0
Ability to tailor the intervention	Adapting the content, intensity, format or delivery of the intervention to participants’ needs, preferences, cultural background or local context increased engagement.	When adaptation was limited by rigid protocols or excessive complexity, staff found it difficult to implement and participants perceived the intervention as less relevant.	Mixed	9	4
Access to participants	Active outreach, clear referral pathways and convenient locations/schedules helped to retain participants.	Physical barriers (e.g. transport, mobility) and lack of outreach in rural or underserved areas limited recruitment and continuity.	Mixed	7	2
Local staff	Involving personnel from the community (e.g. volunteers, community health workers, staff from local organizations) improved trust and cultural appropriateness.	When local staff were insufficiently trained or not well integrated within the interventions, their involvement became unsustainable.	Mixed	7	3
Participant engagement	Regular attendance, active participation and motivation to remain in the program allowed for participants to complete the interventions.	Low motivation, competing responsibilities, or health problems over time led to missed sessions and dropouts.	Mixed	6	5
Staff training and supervision	Initial and ongoing training, guidance and supervision of the people delivering the intervention helped ensure fidelity to protocols, confidence and competence.	Lack of supervision made it difficult to follow protocols and maintain quality over time.	Mixed	5	1
Involvement of health providers	Participation of primary care or mental health professionals (e.g. referrals, co-delivery, monitoring) increased continuity and credibility.	Using conventional methods of referral through medical providers failed to identify socially isolated participants, decreasing access to the intervention.	Mixed	4	1
Perceived benefits	Participants who perceived that the intervention was useful, relevant and improved their symptoms, functioning or quality of life were more likely to remain engaged.	When perceived benefits were unclear or delayed, participants were less motivated to invest time and resources in the program.	Mixed	3	1
Demographic, socioeconomic and cultural characteristics	Incorporating participants’ education, income, ethnicity and cultural beliefs during intervention design helped increase acceptability.	Low literacy, language barriers, stigma, and gender or cultural norms sometimes reduced willingness to seek help and attend group activities.	Mixed	3	3
Trust in staff	Participants’ trust in the staff or volunteers delivering the intervention allowed to ensure confidentiality and safety.	Lack of confidence in staff or stigma around research reduced willingness to share sensitive information.	Mixed	2	1
Use of technology	Access of technologies (e.g. telephone, internet, digital platforms) reduced traveling and supported follow-up.	Low digital literacy or technical problems limited participation.	Mixed	1	1
Duration of intervention		Shorter interventions were insufficient to achieve the benefits of the program, and very long or intensive regimens were perceived as burdensome, increasing dropout.	Barrier	0	3
Self-administered treatments		Materials or activities participants completed on their own (e.g. self-help manuals, self-directed exercises) were often not used as intended, especially among those with cognitive, visual or literacy limitations.	Barrier	0	2
Involvement of family members		Family responsibilities, gatekeeping attitudes or lack of support sometimes limited participants’ time or autonomy to engage in the intervention.	Barrier	0	2

Some determinants mainly acted as facilitators. Financial support by external or institutional funding consistently facilitated recruitment and population access, and provided necessary resources to train staff (Joo et al., 2025; Sadler et al., 2018). Other factors, even though they were mainly facilitators, presented as barriers in a handful of studies. Guaranteeing participant access through convenient locations or active outreach improved engagement and continuity (Guirguis-Younger et al., 2008; Reid et al., 2019), but limited participation in contexts with transport difficulties or limited outreach such as rural or underserved areas (Oh et al., 2020). Partnering with community groups and involving community members during delivery helped programs provide a more accessible and culturally adapted intervention, but hindered delivery when staff were overburdened or poorly integrated with health services (Bartsch & Rodgers, 2009; Rodríguez-Romero et al., 2021; Sirey et al., 2012; Van’t Veer-Tazelaar et al., 2011; Paquet et al., 2023). Staff training and supervision mostly facilitated implementation when initial and ongoing support were available, but in one case unprepared staff changed protocols (Galinha et al., 2023; Kotwal et al., 2021; Sadler et al., 2018). The involvement of health care providers contributed to building trust and accountability, maintaining clinical responsibility and supervision (Guirguis-Younger et al., 2008; Blanchard et al., 1995; Carandang et al., 2020). However, one study identified medical referrals failed to identify socially isolated older adults (Van Citters & Bartels, 2004). Perceived benefits of the programs (e.g. feeling better emotionally or socially) were associated with continued participation (Knight & Alarie, 2017; Mastel-Smith et al., 2007), although benefits were unclear for participants in one study, which increased dropout (Van’t Veer-Tazelaar et al., 2011).

Other determinants were more often described as barriers. Duration of interventions was commonly mentioned as a challenge when programs were experienced as too short to achieve benefits or too long or intensive to be sustained, contributing to dropout (Rodriguez-Romero et al., 2021; Galinha et al., 2023). Self-administered treatments were often not used as intended, particularly by adults with visual or literacy limitations (Guirguis-Younger et al., 2008; Van’t Veer-Tazelaar et al., 2011). The involvement of family members appeared as a barrier, as intervention design could separate family members into different programs and gatekeeping behaviors reduced privacy and opportunities to disclose mental health issues (Knight & Alarie, 2017; Galinha et al., 2023).

Several determinants showed a mixed pattern, operating as facilitators in some studies and as barriers in others. Tailoring interventions to participants’ needs and backgrounds acted as a facilitator when delivery adapted to cultural backgrounds and preferences, but became a barrier when protocols were rigid or adaptation increased complexity beyond what teams could manage (Galinha et al., 2023; Mastel-Smith et al., 2007; Van’t Veer-Tazelaar et al., 2011). Similarly, participant engagement facilitated implementation when participants perceived clear motivations to complete interventions, but acted as a barrier when competing demands and health problems limited attendance (Joo et al., 2025; Reid et al., 2019; Sirey et al., 2012). Incorporating participants’ education, ethnicity and cultural beliefs during design increased acceptability, but language barriers and cultural stigmas around mental health reduced participation (Blanchard et al., 1995; Galinha et al., 2023; Oh et al., 2020). Technology provided tools that boosted engagement in adverse contexts (e.g. COVID pandemic) yet when facing low digital literacy dropouts increased (Kotwal et al., 2021; Paquet et al., 2023). Trust in staff also played a dual role: when present, it ensured participants’ confidentiality and safety, but when stigmas were present it reduced willingness to share sensitive information (Carandang et al., 2020; Joo et al., 2025; Kotwal et al., 2021).

### Methodological Quality of Included Studies

Many authors used comprehensive and validated tools to evaluate mental health outcomes, such as the GDS and PHQ-9, while some studies additionally used structured clinical interviews to establish diagnosis (Sirey et al., 2012, 2021). Several studies were designed as RCT with longitudinal assessments (Blanchard et al., 1995; Galinha et al., 2023; Sirey et al., 2021), and other studies also included qualitative components to capture participant and staff perspectives on the programs (Kotwal et al., 2021; Knight & Alarie, 2017).

At the same time, recurrent methodological limitations were identified using CASP checklists. Several studies had small sample sizes, unreported power or sample size calculations, gender imbalance with samples largely composed of women or lacked control groups (Blanchard et al., 1995; Cuijpers, 1998; Mastel-Smith et al., 2007; Reid et al., 2019; Sirey et al., 2012). Outcomes were measured using self-reported data, which may introduce response and recall bias (Guirguis-Younger et al., 2008) and in some cases, diagnostic tools or criteria used to define depression were limited or inconsistently applied (Sadler et al., 2018; Van Citters & Bartels, 2004). Finally, few studies included long-term follow-up after the interventions ended (Van’t Veer-Tazelaar et al., 2011). These issues should be taken into account when interpreting the effectiveness and implementation findings summarized in this review.

## Discussion

### Summary of Main Findings

The results highlight the potential of interventions delivered in community settings to reduce depressive symptoms and improve mental health and well-being among community-dwelling older adults. Our research identified four main types of interventions, including discussion groups, personal contact, social engagement, skill courses or a combination of one or more of these. Most studies identified a reduction in depressive symptoms, higher remission rates and fewer participants screening positive after interventions, although three studies did not find significant differences when comparing to control groups. The critical appraisal of the included studies highlighted important methodological limitations, including small sample sizes, unreported power calculations, gender imbalance, lack of control groups in some designs, reliance on self-reported outcomes and limited long-term follow-up. These issues mean that the apparent effectiveness of these programs should be interpreted with caution.

Community-based interventions are expected to involve multi-component strategies at both individual and environmental levels, active participation of community members in design and delivery, and an emphasis on empowerment and local needs assessment (Brunton et al., 2017; Castillo et al., 2019; Mikkelsen et al., 2016). Our review shows that interventions to address depressive symptoms in community-dwelling older adults, while often delivered in community settings and sometimes effective, rarely meet all these criteria. Most programs were designed and led by health care professionals, in some cases incorporating community members to deliver interventions. Some elements of community-based approaches were present, such as local staff, gatekeeper models and tailoring to local needs, but community involvement beyond participation and delivery was rare. Although we did not identify interventions adopting explicitly multi-level ecological strategies (e.g. community-wide campaigns or policy advocacy), many programs did involve diverse stakeholders like local organizations, healthcare providers and lay health workers (Schneider et al., 2017). This suggests that current practice lies closer to clinically driven interventions delivered in community settings than to fully community-based models, hence more participatory and structurally oriented approaches could be explored in future work.

Our findings suggest that several implementation determinants should be considered core design features of interventions. Programs that integrate mental health education with social interaction through recreational activities, self-organized social groups, or health education sessions, appear to foster emotional well-being by building social connections (Husaini et al., 1990; Kotwal et al., 2021; Knight & Alarie, 2017; Oh et al., 2020). This combination of education and social engagement addresses emotional and practical aspects of mental health, providing participants with tools they can apply daily (Kotwal et al., 2021; Reid et al., 2019). By using low-cost, self-administered activities, methods with little supervision, or the help of local volunteers, programs can be applicable in locations with limited resources (Guirguis-Younger et al., 2008; Husaini et al., 1990). However, aspects such as financial support, partnering with local volunteers and health care providers, participants’ characteristics and preferences, and adequate staff training are fundamental strategies of community-based interventions, yet in many of the programs included in this review they appeared as contextual afterthoughts rather than explicit design priorities.

Several of the determinants we identified, particularly sociocultural, financial and geographical factors, highlight how context can turn potential facilitators into barriers. Many cultures perceive discussing mental health as shameful; such stigma in mental health poses a challenge to participants’ recruitment and acceptance of mental health care (Blanchard et al., 1995; Joo et al., 2025; Kotwal et al., 2021; Oh et al., 2020). Tailoring interventions to these cultural differences increases participation, but in resource-limited contexts it might overburden teams (Sadler et al., 2018). Technology improves outreach to dispersed and rural areas, where geographical isolation and logistical challenges pose barriers, yet participants’ digital literacy must be considered to decrease access inequities (Kotwal et al., 2021). Hence, context and community preferences and needs should be considered during program design and implementation.

Our review points to several concrete opportunities to strengthen the design and delivery of interventions in community settings. Recruitment challenges were present due to cultural, social, or logistical barriers, such as health problems or scheduling conflicts (Galinha et al., 2023; Joo et al., 2025; Van’t Veer-Tazelaar et al., 2011). Improving accessibility by addressing barriers that hinder participation, offering better transportation options or more flexible scheduling would ensure more consistent participation and improve the reach and impact of these programs (Kotwal et al., 2021; Oh et al., 2020). Furthermore, providing personalized care for participants with severe mental health issues or cognitive impairments would make these interventions more inclusive and effective (Guirguis-Younger et al., 2008; Sirey et al., 2021). Additionally, several studies pointed out the need for better follow-up after discharge to evaluate long-term outcomes (Husaini et al., 1990; Knight & Alarie, 2017; Rodríguez-Romero et al., 2021).

When intervention determinants are taken into consideration, as well as community needs and preferences, programs can intervene early-on in communities’ mental health, especially in areas where traditional healthcare services are deficient. For example, the Atmiyata program in India uses local volunteers who provide mental health support without pay and utilize existing community resources and social capital, enhancing community engagement and empowerment (Joag et al., 2020). Lay Health Workers (LHWs) is another strategy developed in Africa, where community members receive specific training to carry out the interventions, reducing operating costs (Mabunda et al., 2022). Markedly, cultural adaptation enhances the acceptability and effectiveness of these interventions (Mabunda et al., 2022). However, these programs should be seen as complementing, rather than replacing, specialist mental health services, particularly for older adults with complex needs.

### Limitations

This research faced several limitations. Firstly, studies varied widely on the type and intensity of the interventions, the duration of follow-up, and the use of standardized measures. Outcomes were also heterogeneous among studies, mainly due to the absence of a clear consensus on how to screen for depression and measure improvements, and were mainly measured during the course of interventions, with few studies evaluating long-term effects. The age limit that defines older adults also varied between studies. As highlighted by the CASP appraisal, many articles also suffered from methodological limitations such as a small sample size, a lack of a control group, or samples predominantly composed by women, which limits generalizability.

The identification of all relevant studies could have been reduced due to a deficiency in common taxonomy referring to interventions delivered in community settings, clear definitions of community-based programs, and language limitations of the search, only including those spoken by the authors. Publication bias should be considered, as studies with unfavorable outcomes may not have been shared and disseminated.

### Recommendations for Further Research and Practice

The variability in the effectiveness of the interventions highlights the need for more structured and consistently reported approaches, both in program design and in evaluation. Broader literature on community-based mental health programs suggests that these approaches could benefit populations; for example, they have been associated with a reduced likelihood of rehospitalization among Iranian patients with severe mental illness, which is particularly relevant for older adults who often face multiple health problems (Taban et al., 2024). They are also promising strategies to address health outcomes due to their affordability, especially in resource-limited settings, such as the Atmiyata program in India (Joag et al., 2020). In this sense, community-based and community-delivered programs should be seen as complementing, rather than replacing, specialist mental health care.

Several factors could be addressed in future research. We found that most studies included Caucasian or Asian populations, with very few interventions conducted in Latin American settings. Hence, there is an underrepresentation of Latin ethnicities, resulting in difficulties to extrapolate these interventions in Latin communities where older adults experience considerable mental well-being needs and mental-health service provision is limited, fragmented and hamstrung by insufficient resources (Caprioli et al., 2025). Emerging work from Colombia, such as a multi-component psychosocial intervention for older adults in a displaced community in Turbo, begins to address this gap but was not included in the formal review as it was published after our search window (Giebel et al., 2025).

Only a handful of interventions were applied in rural and scattered areas; therefore, more studies should detail the logistical strategies to achieve successful programs in dispersed areas. Additionally, few studies included information regarding the funding and total cost of the interventions. Future studies should include the practical and logistical considerations behind the implementation process to improve replicability. Although several studies reported short-term improvements in depressive symptoms following interventions, evidence on their sustained effects over time remains limited. Future studies should evaluate the maintenance of these effects in the long term.

## Conclusions

Interventions delivered in community settings for older adults show promise for reducing depressive symptoms by offering supportive activities and making use of locally available resources. These programs can help address some of the psychological and social challenges faced by community-dwelling older adults, especially when they are adapted to local needs and preferences. However, their potential is constrained by important limitations in the underlying evidence, including the small number of rigorous randomized trials, frequent reliance on self-reported outcomes, recruitment and retention difficulties, and the practical challenges of tailoring interventions for more vulnerable groups. Addressing these issues in future studies could enhance the effectiveness, equity and sustainability of interventions delivered in community settings, alongside specialist mental health care.

 This paper did not require external funding, except for access to databases, which was covered by the subscriptions of the investigators’ institution.

## Electronic Supplementary Material

Below is the link to the electronic supplementary material.

Supplementary Material 1 (DOCX 2147 KB)

## Appendix 1: Search conducted in four databases

**Table:**

Database	Search equation	Restrictions	Results
PubMed	(((“Aged”[Mesh]) OR (elderly OR “older adults” OR “older people”)) AND ((“Depression”[Mesh] OR “Depressive Disorder”[Mesh] OR “Depressive Disorder,Major”[Mesh]) OR (depres* OR “depressivedisorder”))) AND ((“Community Mental HealthServices”[Mesh]) OR (“community-based intervention”OR “community-based interventions” OR “communityintervention” OR “community interventions”))	None	821
EMBASE	older people':ab,ti OR 'older adults':ab,ti OR 'aged'/expAND'depression'/exp OR 'major depression'/exp OR'depres*':ab,ti OR 'depressive disorder':ab,tiAND'community mental health service'/exp OR 'communitybasedintervention':ab,ti OR 'community-basedinterventions':ab,ti OR 'community intervention':ab,tiOR 'community interventions':ab,ti	None	173
Ovid	exp Aged/OR (elderly or “older people” or “olderadults”).ab,ti.ANDexp Depression/ or exp Depressive Disorder/ or expDepressive Disorder, Major/ OR (depres* or “depressivedisorder”).ab,ti.ANDexp Community Mental Health Services/ OR(“community-based intervention” or “community-basedinterventions” or “community intervention” or"community interventions”).ab,ti.	None	94
PsycNet	MeSH: Aged OR elderly OR “older people” OR “olderadults”MeSH: Depression OR MeSH: Depressive Disorder ORMeSH: Depressive Disorder, Major OR depres*MeSH: Community Mental Health Services OR“community-based intervention” OR “community-based interventions” OR “community intervention” OR“community interventions”	None	21

## References

[CR1] Bartsch, D. A., & Rodgers, V. K. (2009). Senior reach outcomes in comparison with the Spokane Gatekeeper program. *Care Management Journals*, *10*(3), 82–88. 10.1891/1521-0987.10.3.82 19772205

[CR2] Blanchard, M. R., Waterreus, A., & Mann, A. H. (1995). The effect of primary care nurse intervention upon older people screened as depressed. *International Journal of Geriatric Psychiatry*. 10.1002/gps.930100405 10479744

[CR3] Brasileiro, L. E. E., Dantas, A. A. G., Linhares, D. B., Vale, H. A., Terradas-Monllor, M., Ochandorena-Acha, M., Paiva, A. L. M., de Medeiros, M. Y. D., Jerez-Roig, J., & de Souza, D. L. B. (2024). Incidence of depression among community-dwelling older adults: A systematic review. *Psychogeriatrics: The Official Journal of the Japanese Psychogeriatric Society*, *24*(2), 496–512. 10.1111/psyg.13081 38263357 PMC11578035

[CR4] Brunton, G., Thomas, J., O’Mara-Eves, A., Jamal, F., Oliver, S., & Kavanagh, J. (2017). Narratives of community engagement: A systematic review-derived conceptual framework for public health interventions. *BMC Public Health*, *17*(1), 944. 10.1186/s12889-017-4958-4 29228932 PMC5725895

[CR5] Cai, H., Jin, Y., Liu, R., Zhang, Q., Su, Z., Ungvari, G. S., Tang, Y. L., Ng, C. H., Li, X. H., & Xiang, Y. T. (2023). Global prevalence of depression in older adults: A systematic review and meta-analysis of epidemiological surveys. *Asian Journal of Psychiatry*, *80*, 103417. 10.1016/j.ajp.2022.103417 36587492

[CR6] Caprioli, T., Zuluaga-Callejas, M. I., Gabbay, M., Saldarriaga-Ruiz, G., Castaño-Pineda, Y., Montoya, E. M., Robertson, A., & Giebel, C. (2025). Mental health has been left behind: A qualitative exploration of stakeholders’ perceptions of older adults’ mental well-being needs and services in a Colombian displaced community. *Clinical Gerontologist*, *48*(5), 1282–1293. 10.1080/07317115.2025.2467921 39954012

[CR7] Carandang, R. R., Shibanuma, A., Kiriya, J., Vardeleon, K. R., Asis, E., Murayama, H., & Jimba, M. (2020). Effectiveness of peer counseling, social engagement, and combination interventions in improving depressive symptoms of community-dwelling Filipino senior citizens. *PLoS ONE*, *15*(4), e0230770. 10.1371/journal.pone.0230770 32236104 PMC7112231

[CR8] Castillo, E. G., Ijadi-Maghsoodi, R., Shadravan, S., Moore, E., Mensah, M. O. III, Docherty, M., Aguilera Nunez, M. G., Barcelo, N., Goodsmith, N., Halpin, L. E., Morton, I., Mango, J., Montero, A. E., Koushkaki, R., Bromley, S., Chung, E., Jones, B., Gabrielian, F., Gelberg, S., Greenberg, L., & Wells, J. M., K. B (2019). Community interventions to promote mental health and social equity. *Current Psychiatry Reports*, *21*(5), 35. 10.1007/s11920-019-1017-0 30927093 PMC6440941

[CR9] Corpas, J., Gilbody, S., & McMillan, D. (2022). Cognitive, behavioural or cognitive-behavioural self-help interventions for subclinical depression in older adults: A systematic review and meta-analysis. *Journal of Affective Disorders*, *308*, 384–390. 10.1016/j.jad.2022.04.085 35460732

[CR11] Critical Appraisal Skills Programme. (2023). CASP Checklists. https://casp-uk.net/casp-tools-checklists/

[CR10] Cuijpers, P. (1998). Psychological outreach programmes for the depressed elderly: a meta-analysis of effects and dropout. *International journal of geriatric psychiatry*, *13*(1), 41–48. 10.1002/(SICI)1099-1166(199801)13:1<41::AID-GPS729>3.0.CO;2-B 9489580

[CR12] Galinha, I. C., Fernandes, H. M., Lima, M. L., & Palmeira, A. L. (2023). Intervention and mediation effects of a community-based singing group on older adults’ perceived physical and mental health: the Sing4Health randomized controlled trial. *Psychology & health*, *38*(1), 73–93. 10.1080/08870446.2021.1955117 34355628

[CR13] Gardiner, C., Geldenhuys, G., & Gott, M. (2018). Interventions to reduce social isolation and loneliness among older people: an integrative review. *Health & social care in the community*, *26*(2), 147–157. 10.1111/hsc.12367 27413007

[CR14] Giebel, C., Shrestha, N., Reilly, S., White, R. G., Zuluaga, M. I., Saldarriaga, G., Liu, G., Allen, D., & Gabbay, M. (2022). Community-based mental health and well-being interventions for older adults in low- and middle-income countries: A systematic review and meta-analysis. *BMC Geriatrics*, *22*(1), 773. 10.1186/s12877-022-03453-1 36175867 PMC9520120

[CR15] Giebel, C., Montoya, E., Saldarriaga, G., Caprioli, T., Gabbay, M., Martinez, D., Rua, J., & Zuluaga, M. I. (2025). Addressing unmet mental health needs of older adults in Turbo, Colombia: A multi-component psychosocial intervention feasibility study. *International Journal for Equity in Health*, *24*(1), 21. 10.1186/s12939-025-02381-x 39833867 PMC11748288

[CR16] Guirguis-Younger, M., Cappeliez, P., & Younger, A. (2008). A community-based intervention for treating depression in seniors. *The Canadian Journal of Nursing Research = Revue canadienne de recherche en sciences infirmieres*, *40*(1), 61–79. 18459272

[CR17] Haddaway, N. R., Page, M. J., Pritchard, C. C., & McGuinness, L. A. (2022). PRISMA2020: An R package and Shiny app for producing PRISMA 2020-compliant flow diagrams, with interactivity for optimised digital transparency and Open. *Synthesis Campbell Systematic Reviews*, *18*, e1230. 10.1002/cl2.1230 36911350 PMC8958186

[CR18] Han, F-F., Wang, H-X., Wu, J-J., Yao, W., Hao, C-F., & Pei, J-J. (2021). Depressive symptoms and cognitive impairment: A 10-year follow-up study from the Survey of Health, Ageing and Retirement in Europe. *European Psychiatry*, *64*(1), e55. 10.1192/j.eurpsy.2021.2230 34446123 PMC8446071

[CR20] Hu, T., Zhao, X., Wu, M., Li, Z., Luo, L., Yang, C., & Yang, F. (2022). Prevalence of depression in older adults: A systematic review and meta-analysis. *Psychiatry Research*, *311*, 114511. 10.1016/j.psychres.2022.114511 35316691

[CR21] Husaini, B. A., Castor, R. S., Whitten-Stovall, R., Moore, S. T., Neser, W., Linn, J. G., & Griffin, D. (1990). An evaluation of a therapeutic health program for the black elderly. *Journal of Health & Social Policy*, *2*(2), 67–85. 10.1300/j045v02n02_05 10111763

[CR19] Jalali, A., Ziapour, A., Karimi, Z., Rezaei, M., Emami, B., Kalhori, R. P., Khosravi, F., Sameni, J. S., & Kazeminia, M. (2024). Global prevalence of depression, anxiety, and stress in the elderly population: A systematic review and meta-analysis. *BMC Geriatrics*, *24*(1), 809. 10.1186/s12877-024-05311-8 39367305 PMC11451041

[CR22] Joag, K., Shields-Zeeman, L., Kapadia-Kundu, N., Kawade, R., Balaji, M., & Pathare, S. (2020). Feasibility and acceptability of a novel community-based mental health intervention delivered by community volunteers in Maharashtra, India: The Atmiyata programme. *BMC Psychiatry*, *20*(1), 48. 10.1186/s12888-020-2466-z 32028910 PMC7006077

[CR23] Joo, J. H., Xie, A., Choi, N., Gallo, J. J., Zhong, Y., Ma, M., Locascio, J. J., Khemraj, U., Mace, R. A., & Solomon, P. (2025). A mixed methods effectiveness study of a peer support intervention for older adults during the COVID-19 pandemic: Results of a randomized clinical trial. *The American Journal of Geriatric Psychiatry: Official Journal of the American Association for Geriatric Psychiatry*, *33*(4), 389–401. 10.1016/j.jagp.2024.09.013 39438236 PMC11875900

[CR24] Knight, C. A., & Alarie, R. M. (2017). Improving mental health in the community: Outcome evaluation of a geriatric mental health day treatment service. *Clinical Gerontologist*, *40*(2), 77–87. 10.1080/07317115.2016.1263709 28452673

[CR25] Kotwal, A. A., Fuller, S. M., Myers, J. J., Hill, D., Tha, S. H., Smith, A. K., & Perissinotto, M., C (2021). A peer intervention reduces loneliness and improves social well-being in low-income older adults: A mixed-methods study. *Journal of the American Geriatrics Society*, *69*(12), 3365–3376. 10.1111/jgs.17450 34449870 PMC8648986

[CR26] Mabunda, D., Oliveira, D., Sidat, M., Cavalcanti, M. T., Cumbe, V., Mandlate, F., Wainberg, M., Cournos, F., & de Jesus Mari, J. (2022). Cultural adaptation of psychological interventions for people with mental disorders delivered by lay health workers in Africa: Scoping review and expert consultation. *International Journal of Mental Health Systems*, *16*(1), 14. 10.1186/s13033-022-00526-x 35168650 PMC8845308

[CR27] Mastel-Smith, B. A., McFarlane, J., Sierpina, M., Malecha, A., & Haile, B. (2007). Improving depressive symptoms in community-dwelling older adults: A psychosocial intervention using life review and writing. *Journal of Gerontological Nursing*, *33*(5), 13–19. 10.3928/00989134-20070501-04 17511331

[CR28] Mikkelsen, B. E., Novotny, R., & Gittelsohn, J. (2016). Multi-level, multi-component approaches to community based interventions for healthy living—a three case comparison. *International Journal of Environmental research and Public Health*, *13*(10), 1023. 10.3390/ijerph13101023 27775630 PMC5086762

[CR29] Motamed-Jahromi, M., & Kaveh, M. H. (2021). Effective interventions on improving elderly’s independence in activity of daily living: A systematic review and logic model. *Frontiers in Public Health*, *8*, 516151. 10.3389/fpubh.2020.516151 33659228 PMC7917261

[CR30] Nguyen, D., & Vu, C. M. (2013). Current depression interventions for older adults: A review of service delivery approaches in primary care, home-based, and community-based settings. *Current Translational Geriatrics and Experimental Gerontology Reports*, *2*(1), 37–44.

[CR31] Oh, I. M., Cho, M. J., Hahm, B. J., Kim, B. S., Sohn, J. H., Suk, H. W., Jung, B. Y., Kim, H. J., Kim, H. A., Choi, K. B., You, D. H., Lim, A. R., Park, I. O., Ahn, J. H., Lee, H., Kim, Y. H., Kim, M. R., & Park, J. E. (2020). Effectiveness of a village-based intervention for depression in community-dwelling older adults: A randomised feasibility study. *BMC Geriatrics*, *20*(1), 89. 10.1186/s12877-020-1495-2 32131745 PMC7057500

[CR32] Paquet, C., Whitehead, J., Shah, R., Adams, A. M., Dooley, D., Spreng, R. N., Aunio, A. L., & Dubé, L. (2023). Social Prescription Interventions Addressing Social Isolation and Loneliness in Older Adults: Meta-Review Integrating On-the-Ground Resources. *Journal of medical Internet research*, *25*, e40213. 10.2196/40213 37195738 PMC10233446

[CR33] Peters, M., Godfrey, C., McInerney, P., Munn, Z., Tricco, A., & Khalil, H. (2020). Scoping reviews. En *JBI manual for evidence synthesis*. JBI. 10.11124/JBIES-20-00167 33038124

[CR34] Porteny, T., Alegría, M., Del Cueto, P., Fuentes, L., Markle, S. L., NeMoyer, A., & Perez, G. K. (2020). Barriers and strategies for implementing community-based interventions with minority elders: Positive minds-strong bodies. *Implementation Science Communications*, *1*, 41. 10.1186/s43058-020-00034-4 32885198 PMC7427860

[CR35] Reid, K. F., Laussen, J., Bhatia, K., Englund, D. A., Kirn, D. R., Price, L. L., Manini, T. M., Liu, C. K., Kowaleski, C., & Fielding, R. A. (2019). Translating the lifestyle interventions and independence for elders clinical trial to older adults in a real-world community-based setting. *The Journals of Gerontology Series A: Biological Sciences and Medical Sciences*, *74*(6), 924–928. 10.1093/gerona/gly152 30010808 PMC6521918

[CR36] Reynolds, C. F. III, Jeste, D. V., Sachdev, P. S., & Blazer, D. G. (2022). Mental health care for older adults: Recent advances and new directions in clinical practice and research. *World Psychiatry: Official Journal of the World Psychiatric Association (WPA)*, *21*(3), 336–363. 10.1002/wps.20996 36073714 PMC9453913

[CR37] Rodríguez-Romero, R., Herranz-Rodríguez, C., Kostov, B., Gené-Badia, J., & Sisó-Almirall, A. (2021). Intervention to reduce perceived loneliness in community-dwelling older people. *Scandinavian Journal of Caring Sciences*, *35*(2), 366–374. 10.1111/scs.12852 32285499

[CR38] Sadler, P., McLaren, S., Klein, B., Harvey, J., & Jenkins, M. (2018). Cognitive behavior therapy for older adults with insomnia and depression: A randomized controlled trial in community mental health services. *Sleep*. 10.1093/sleep/zsy104 29800468

[CR39] Schneider, S., Diehl, K., Görig, T., Schilling, L., De Bock, F., Hoffmann, K., Albrecht, M., Sonntag, D., & Fischer, J. (2017). Contextual influences on physical activity and eating habits—options for action on the community level. *Bmc Public Health*. 10.1186/s12889-017-4790-x 28964266 PMC5622514

[CR42] Sirey, J. A., Hannon, C. P., D’Angelo, D., & Knies, K. (2012). A community treatment intervention advancing active treatment in the elderly (ACTIVATE): A pilot study. *Journal of Gerontological Social Work*, *55*(5), 382–391. 10.1080/01634372.2011.644029 22783956 PMC3397399

[CR43] Sirey, J. A., Solomonov, N., Guillod, A., Zanotti, P., Lee, J., Soliman, M., & Alexopoulos, G. S. (2021). PROTECT: A novel psychotherapy for late-life depression in elder abuse victims. *International Psychogeriatrics*, *33*(5), 521–525. 10.1017/S1041610221000430 33926591 PMC8169597

[CR40] Sivertsen, H., Bjørkløf, G. H., Engedal, K., Selbæk, G., & Helvik, A. S. (2015). Depression and quality of life in older persons: A review. *Dementia and Geriatric Cognitive Disorders*, *40*(5–6), 311–339. 10.1159/000437299 26360014

[CR41] Sjöberg, L., Karlsson, B., Atti, A. R., Skoog, I., Fratiglioni, L., & Wang, H. X. (2017). Prevalence of depression: Comparisons of different depression definitions in population-based samples of older adults. *Journal of Affective Disorders*, *221*, 123–131. 10.1016/j.jad.2017.06.011 28645024

[CR44] Taban, M., Nooraeen, S., Tanha, K., Moradi-Lakeh, M., & Malakouti, S. K. (2024). Effectiveness and cost-effectiveness of community-based mental health services for individuals with severe mental illness in Iran: A systematic review and meta-analysis. *BMC Psychiatry*, *24*(1), 256. 10.1186/s12888-024-05666-7 38575916 PMC10993444

[CR45] Tricco, A. C., Lillie, E., Zarin, W., O’Brien, K. K., Colquhoun, H., Levac, D., Moher, D., Peters, M. D. J., Horsley, T., Weeks, L., Hempel, S., Akl, E. A., Chang, C., McGowan, J., Stewart, L., Hartling, L., Aldcroft, A., Wilson, M. G., Garritty, C., Lewin, S., ... Straus, S. E. (2018). PRISMA extension for scoping reviews (PRISMA-ScR): Checklist and explanation. *Annals of Internal Medicine*, *169*, 467–473. 10.7326/M18-0850 30178033

[CR46] United Nations (n. d.). *Global issues: Ageing.* Retrieved from https://www.un.org/en/global-issues/ageing

[CR47] Van Citters, A. D., & Bartels, S. J. (2004). A systematic review of the effectiveness of community-based mental health outreach services for older adults. *Psychiatric Services (Washington, DC)*, *55*(11), 1237–1249. 10.1176/appi.ps.55.11.1237 15534012

[CR48] van’t Veer-Tazelaar, N., van Marwijk, H., van Oppen, P., van der Horst, H., Smit, F., Cuijpers, P., & Beekman, A. (2011). Prevention of late-life anxiety and depression has sustained effects over 24 months: A pragmatic randomized trial. *The American Journal Of Geriatric Psychiatry : Official Journal Of The American Association For Geriatric Psychiatry*, *19*(3), 230–239. 10.1097/JGP.0b013e3181faee4d 21425519

