# Supplementary material for: Interventions to Improve Depressive Symptoms in Community-Dwelling Older Adults: A Scoping Review
Source: J Prev (2022). 2026 May 28;47(5):819–50. doi: 10.1007/s10935-026-00911-z (PMC13582149; doi:10.1007/s10935-026-00911-z)
Supplement: Supplementary file 1 [file 10935_2026_911_MOESM1_ESM.docx]

**Supplementary Table S1**. Implementation determinants by study

| **Type of intervention** | **Study ID** | **Implementation determinant categories** | **Description of determinants (examples)** |
| --- | --- | --- | --- |
| Personal contact | Sirey, 2021 | - Local staff (+)  - Ability to tailor the intervention (+)  - Participant engagement (+)  - Demographic, socioeconomic and cultural characteristics (-) | The intervention was developed in partnership with community organizations.  The program was specifically designed for abuse victims and could be customized to meet their needs.  Every participant in the intervention group began therapy, surpassing the pre-established standard of 75% for feasibility.  The study was conducted only in English, potentially excluding non-English speakers. |
|  | Cuijpers, 1998 | None reported | None reported |
|  | Blanchard, 1995 | - Ability to tailor the intervention (+)  - Involvement of health providers (+)  - Staff training and supervision (+)  - Participant engagement (-) | Intervention plans were generated individually for each participant.  The participants' general practitioners maintained clinical responsibility, supervising decisions made.  The study nurse maintained contact with the psychiatry team and could consult them at any time.  Nearly half of the activities were not implemented because of patient refusal. |
|  | Kotwal, 2021 | - Financial support (+)  - Ability to tailor the intervention (+)  - Demographic, socioeconomic and cultural characteristics (+)  - Staff training and supervision (+)  - Trust in staff (+)  - Use of technology (-) | Funding from local jurisdiction allowed to sustain the program.  The intervention was inclusive on diverse backgrounds and reduced barriers in recruitment.  Participants included were from a wide array of ethnicities due to a broad inclusion criteria.  Staff received training before and while the intervention was carried out.  Participants and staff were matched according to common interests, facilitating trust.  Covid-19 shelter-in-place orders forced to change the dynamics of the intervention switching to phone calls with the loss of 19% of participants. |
|  | Joo, 2025 | - Financial support (+)  - Local staff (+)  - Trust in staff (-)  - Access to participants (-)  - Participant engagement (-) | There was strong governmental backing, which facilitated the recruitment of peer mentors.  Community organizations helped distribute information and engage participants.  Recruitment faced challenges because of the stigma associated with mental health, as well as distrust and unfamiliarity with research.  The transition to remote healthcare services during COVID-19 made it difficult to recruit participants from primary care clinics.  The population presented difficulties in maintaining engagement and retention, largely due to stigma and distrust. |
| Discussion groups | Sadler, 2018 | - Staff training and supervision (+)  - Financial support (+)  - Ability to tailor the intervention (-)  - Duration of intervention (-) | Inexperienced professionals were trained to deliver the intervention.  The program used professionals already linked to the mental health services and did not require to hire more staff.  One of the interventions was too advanced and complicated to practice; a simpler treatment may be better suited.  One of the interventions required a longer course of therapy and a more specialized training package. |
|  | Mastel-Smith, 2007 | - Perceived benefit (+)  - Ability to tailor the intervention (-) | Participants felt an overall improvement in their well-being as a result of their involvement.  The workshop required participants to have a fundamental level of literacy. |
|  | Husaini, 1990 | None reported | None reported |
| Social engagement | Galinha, 2023 | - Perceived benefit (+)  - Demographic, socioeconomic and cultural characteristics (+)  - Ability to tailor the intervention (+)  - Involvement of family members (-)  - Duration of intervention (-)  - Staff training and supervision (-) | The positive effect experienced during the intervention led to increased well-being and high participation rates.  Inclusion criteria allowed the recruitment of a diverse community-based elderly sample.  The program included traditional songs selected by the team and the participants after discussion.  Many participants that had enrolled with friends or spouses were lost during follow-up.  The duration of the intervention may have been insufficient to improve physical and mental health outcomes.  The team (pianist and maestro) shortened the group dynamic's exercises, which were expected to promote social and physical/mental benefits. |
|  | Paquet,  2023 | - Use of technology (+)  - Participant engagement (+)  - Access to participants (+)  - Local staff (-)  - Ability to tailor the intervention (-) | Interventions included technology training and networking for older adults, plus befriending by phone/video and radio-based programs.  Interventions worked better when there was a shared purpose and participants felt autonomous.  Social prescribing effectively links primary care patients to community, nonmedical resources.  Patient care is fragmented across primary care, community and public health care, limiting coordination and restricting access to services.  Without a unified system to identify and classify services, adapting these programs across contexts is difficult. |
| Skill course | Sirey, 2012 | - Ability to tailor the intervention (+)  - Participant engagement (+)  - Local staff (-) | Most participants defined a plan that would strengthen their previous depression treatment.  By setting personalized goals and solutions, the intervention empowers older adults in the management of their depression treatment.  Identifying available referral resources is necessary before delivering the intervention, which may not be available to all communities. |
| Discussion groups and personal contact | Oh, 2020 | - Local staff (+)  - Participant engagement (+)  - Demographic, socioeconomic and cultural characteristics (-)  - Access to participants (-) | Local volunteers were responsible for monitoring the at-risk older adults.  Participants were guided to assume active rolls to strengthen their autonomy.  Boundaries in Korean culture to disclose mental health problems to other people because it is perceived negatively.  Lack of medical services in rural areas hinders general physician-based screening-programs. |
|  | Van Citters, 2004 | - Access to participants (+)  - Local staff (+)  - Involvement of health providers (-) | Nontraditional outreach programs (at-home, community centers) improve access to interventions.  Utilizing community figures (e.g., utility workers) reaches older adults who may not access services through traditional health systems.  Conventional methods of referral through medical providers often fail to identify socially isolated older adults. |
| Skill course and personal contact | Van 't Veer-Tazelaar, 2011 | - Local staff (+)  - Ability to tailor the intervention (+)  - Participant engagement (+)  - Self-administered treatments (-)  - Perceived benefit (-) | The implementation was done in collaboration with local home care agencies, regional mental health organizations, and general practitioners.  The intervention follows a stepped care model, which is structured in phases based on the participant's progress.  The program aims to empower individuals through personalized education, counseling, and regular engagement with their symptom levels.  The self-help course may backfire by making participants more aware of their limitations, which could be discouraging.  The nature and volume of questions might be overwhelming or distressing for participants, leading to potential dropouts. |
|  | Knight, 2017 | - Perceived benefit (+)  - Participant engagement (-)  - Ability to tailor the intervention (-)  - Involvement of family members (-) | Participants quickly developed a bond with other group members due to the common experience of addressing their mental health challenges.  Dropouts were often due to unexpected issues such as health concerns, transportation problems, or vacations.  Some participants indicated their long-standing mental health issues required more intensive and individualized treatment.  Participants generally did not feel comfortable including family members in the program's formal processes. |
|  | Guirguis-Younger, 2008 | - Ability to tailor the intervention (+)  - Involvement of health providers (+)  - Access to participants (+)  - Participant engagement (+)  - Self-administered treatments (-) | Self-administered treatment can be adjusted based on participants’ schedules and needs.  A community of nurses implemented the intervention at a minimal cost, both financially and timewise.  Self-administered interventions with minimal contact facilitated participants to follow the activities.  Home-based approach is unlikely to carry the stigma associated with seeking mental health services.  Self-applied treatments might be biased in both implementation and progress reporting, even with the supervision. |
|  | Rodríguez-Romero, 2021 | - Local staff (+)  - Ability to tailor the intervention (+)  - Access to participants (+)  - Involvement of health providers (+)  - Participant engagement (-)  - Duration of the intervention (-) | Staff included neighborhood civil, social, and religious organizations.  The intervention can be replicated in other regions, adapting the activities provided according to neighborhood resources.  Follow-up could be done in home or health center appointments at the patient’s convenience.  The intervention was led by a nurse practitioner and carried out by primary healthcare teams and other community agents.  Potential participants were excluded from the study due to competing responsibilities and lack of time.  Most participants did not attend all activities due to the prolonged nature of the intervention. |
| Multicomponent | Carandang, 2020 | - Trust in staff (+)  - Involvement of health providers (+)  - Staff training and supervision (+)  - Access to participants (+) | Counselors were senior volunteers from the community who shared demographic characteristics with the participants.  The involvement of health providers may have increased quality and accountability.  Peer counselors underwent a 40-hour training for leadership and peer counseling.  As the intervention takes place in a small geographical venue, it is easier to follow up participants' progress. |
|  | Reid, 2019 | - Access to participants (+)  - Demographic, socioeconomic and cultural characteristics (+)  - Participant engagement (-)  - Local staff (-) | Using an existing community outreach partnership facilitated access to a senior center and its resources (e.g. transportation, wellness activities).  Minimal exclusion criteria allowed a more inclusive recruitment of older adults.  The adherence rate was lower than other community-based exercise programs.  The intervention was delivered by university-based exercise physiologists, instead of training existing community-based personnel. |
|  | Bartsch, 2009 | - Local staff (+)  - Access to participants (+)  - Ability to tailor the intervention (+)  - Staff training and supervision (+)  - Demographic, socioeconomic and cultural characteristics (-) | The program trained community members, which referred seniors for the intervention.  Staff included nontraditional referral sources who were members of the community that had contact with seniors in community-based environments.  A call center was included to gather information of the participants and provide in-home assessments.  Cases with special concerns were reviewed in clinical supervision.  Participants of the program were already enrolled in a mental health program, which may have limited the effectiveness of the intervention. |
